# Bispecific antibodies in indolent B-cell lymphomas

**DOI:** 10.3389/fimmu.2023.1295599

**Published:** 2024-01-11

**Authors:** Vivek S. Radhakrishnan, Andrew J. Davies

**Affiliations:** ^1^ Cancer Care Group, Division B, University Hospital of Southampton National Health Service (NHS) Trust, Southampton, United Kingdom; ^2^ School of Cancer Sciences, University of Southampton, Southampton, United Kingdom

**Keywords:** lymphoma, non-Hodgkin lymphoma, indolent lymphoma, immunotherapy, bi-specific antibodies, antibody therapies

## Abstract

The advent of immunotherapy in lymphomas, beginning with Rituximab, have led to paradigm shifting treatments that are increasingly bringing a greater number of affected patients within the ambit of durable disease control and cure. Bispecific antibodies harness the properties of the immunoglobulin antibody structure to design molecules which, apart from engaging with the target tumour associated antigen, engage the host’s T-cells to cause tumour cell death. Mosunetuzumab, an anti-CD20 directed bispecific antibody was the first to be approved in follicular lymphoma, this has now been followed by quick approvals of Glofitamab and Epcoritamab in diffuse large B-cell lymphomas. This article reviews contemporary data and ongoing studies evaluating the role of bispecific antibodies in indolent b-cell non Hodgkin lymphomas. This is an area of active research and presents many opportunities in advancing the treatment of indolent lymphomas and potentially forge a chemo-free treatment paradigm in this condition.

## Introduction

Köhler and Milstein’s outstanding work on the hybridoma technology set in motion monoclonal antibody based biomedical research, which has led to the development of landmark diagnostic and therapeutic platforms (1). The success of rituximab, a naked anti-CD20 monoclonal antibody, in the treatment of B-cell lymphomas is an important milestone in the use of immunotherapy in cancer (2). Immunotherapy based strategies have enabled paradigm shifting treatment approaches in several cancer subtypes, the regulatory approval and rapid acceptance of checkpoint inhibitors and Chimeric Antigen Receptor-T cell (CAR-T) therapy being the most noteworthy. Monoclonal antibody based treatments work on multiple effector functions dependent on the target, which may range from passive immunity against a target antigen that is strongly associated with a tumour, to an immunostimulatory function triggered by the stimulation or inhibition of receptors on immune-effector cells. When a monoclonal antibody engages with the target antigen there is an Fc-gamma receptor (FcγR) triggered mobilisation of cytotoxic and phagocytic host immune cells (3). Beyond this antibody dependant cell cytotoxicity, other mechanisms like complement dependant cytotoxicity, signalling-induced apoptosis, etc. have also been suggested, and these have been reviewed elsewhere (3).

In recent times, immunotherapeutic interventions have led to a goldrush of opportunities in the treatment of cancers and other disease conditions. Apart from naked monoclonal antibodies which are now routinely used, antibody-drug conjugates (ADC), i.e. monoclonal antibody molecules coupled with a drug/toxin payload, have made their mark in lymphomas, e.g. Brentuximab vedotin in Hodgkin Lymphoma, Polatuzumab vedotin and Loncastuximab teserine in diffuse large B-cell Lymphomas (4, 5). Chimeric antigen receptor- T (CAR-T) cell therapy uses T-cells, re-engineered to express a designer T-cell receptor (TCR) against a lymphoma directed antigen like CD19 to clinical benefit from tumour cell lysis independent of major histocompatibility (MHC) restricted T-cell receptor activation of T-cells (6). CAR-T therapies have now become standard of care in the setting of relapsed diffuse large B-cell lymphomas (DLBCL), mantle cell lymphoma (MCL) and b-cell acute lymphoblastic leukaemia (7–9).

### Bispecific antibodies

Bispecific antibodies (BsA) are immunoglobulin (Ig) molecules or their derivatives, which beyond targeting an antigen of interest also engage a host immune cell, e.g. T-cells, to trigger immune mediated direct cell cytotoxicity or phagocytosis (10). The clinical use of T-cell engaging antibodies in B-cell neoplasms, began with the use of Blinatumomab [an anti-CD19 bispecific antibody in acute lymphoblastic leukaemia (11). BsA are now maturing into a new treatment frontier in lymphomas, with recent US-FDA approvals of Glofitamab and Epcoritamab in relapsed or refractory (R/R) DLBCL and Mosunetuzumab in R/R Follicular lymphoma (FL) (12–14). This short review will focus on the role of Bispecific monoclonal immune cell engaging antibodies in indolent (low grade) B-cell non-Hodgkin Lymphomas (B-NHL).

## Structure and function of bispecific antibodies

Bispecific antibodies (BsA) are assembled either in a structure similar to an Ig molecule replete with an Fc (fragment crystallizable) moiety, or by fusing individual antigen binding sites as dual affinity redirecting antibodies (Bispecific T-cell engagers, BiTE^®^). Most BsA in development and/or use in lymphomas have an IgG like structure, whereas Blinatumomab is an apt example of a BiTE molecule. Different technologies employed over time have facilitated the development of a robust and promising pipeline of BsA molecules in clinical trials. The field has now further expanded, with the development of multi-specific (multi-antigen directed) antibodies and Ig like fusion proteins, adding to the panoply of available molecules. A detailed review of the structure, function, technology, and mechanisms of action of BsA can be found elsewhere (15).

Once the BsA engages with target antigens on the tumour cell and immune effector cell [T-cell, Natural Killer (NK)-cell, macrophages], there is activation of the immune effector cell resulting in tumour cell lysis in an MHC independent mechanism (15). Immune cell engagement occurs within and potentially outside the tumour. With release of cytokines in the milieu, an inflammatory milieu is triggered which acts locally possibly setting off a bystander effect on the tumour microenvironment apart from a systemic response (16). Multiple factors determine the uptake and pharmacodynamic interactions of BsA, which include (but not limited to) the following: cell surface expression and stability of target antigens, structure and antigen affinity of the antibody, molecular size and flexibility of antibody, ease and avidity of immune-synapse formation, expression and activation of co-stimulatory or co-inhibitory receptors on the engaged immune effector cells, cell cytotoxicity, immune cell dynamics in different body compartments, etc. (17). Given these unique properties, BsA dosing and dose-modelling are not based on conventional chemotherapy or naked monoclonal antibody/ADC based dose-models, and rely on biologically effective dosing. Most BsA follow a fixed flat dosing schedule after an initial course of step-up dosing to accommodate for cytokine release syndrome, tumour lysis and immune response (18–21).

Among multiple B-cell tumour antigens, CD20 and CD19 targeting BsA molecules are frontrunners in development, potentially due to their higher tumour cell cytotoxicity (22, 23). **Table 1** lists the molecules that have been approved or are in development in indolent B-cell Lymphomas. Among these and as on date, Mosunetuzumab (Lunsumio^®^) has been granted accelerated and conditional approval by the US-FDA and EMA, respectively, for patients with relapsed follicular lymphoma after two or more lines of therapy (24).

**Table 1:** Candidate bispecific monoclonal antibody (BsA) molecules.

Drug Name	Structural details	CDXX : CD3 ratio	Route of administration	Approved or RP2D dose	Manufacturer
Mosunetuzumab	IgG1, CD20xCD3 BsA	1:1	Intravenous	30mg [Step-up: 1mg/2/60/60/30mg]	Roche^®^
Glofitamab	IgG1, CD20xCD3 BsA	2:1	Intravenous	30mg Cycle 1: 2.5mg>10mg Cycle 2+: 30mg	Roche^®^
Epcoritamab	IgG1, CD20xCD3 BsA	1:1	Subcutaneous	48mg Cycle 1: 0.16mg > 8mg > 48mg	Genmab^®^
Odronextamab	IgG4, CD20xCD3 BsA	1:1	Intravenous	*80mg (Follicular Lymphoma)/* *160mg (DLBCL), ongoing*	Regeneron^®^
Plamotamab	IgG1, CD20xCD3 BsA	1:1	Intravenous	*20/50mg, ongoing*	Xencor^®^
IgM-2323 (Imvotamab)	IgM, CD20xCD3 BsA	10:1	Intravenous	*100/300mg, ongoing*	IGM Biosciences^®^
TNB-486	IgG4, **CD19x**CD3 BsA	1:1	Intravenous	NR	AstraZeneca^®^
IMM0306	IgG1, CD20x**SIRPα(CD47)** BsA	1:1	Intravenous	NR	ImmuneOnco^®^
RO7227166, (Englumafusp alpha)	**CD19x4-1BB** BsAb like *fusion protein*	NR	Intravenous	NR	Roche^®^
GNC-038 (Emfizatamab)	IgG-scFv-conjugated tetraspecific antibody (**αCD19x**CD3x4–1BBxPD-L1)	NR	Intravenous	NR	Systimmune^®^

## Clinical outcomes in early phase studies of indolent B-NHL

**Table 2** lists the significant studies (including abstracts) using BsA in indolent B-cell Lymphomas.

**Table 2:** Published studies and abstracts evaluating bispecific molecules in indolent B-cell lymphomas.

Author	Year	Type of study	No. of pts	Median prior lines of Rx	Regimen	Percentage Prior AHCT/CAR-T	Median cycles	Response			Grade III-IV Tox, %	Comments
								ORR (%)	CR (%)	PR (%)		
Budde et al (25)	2022	Phase 1/2	68 [FL=65, MZL=2, SLL=1]	3	**Mosunetuzumab IV**	AHCT (18)/CAR-T (6)	5 (1-17)	66.2	48.5	–	Neutropenia:25.4 Hypophosphatemia:15 FN: 3.6, CRS: 1	NCT02500407 TAE discontinuation 3.6% Median PFS= 11.8m
Budde et al (26)	2022	Phase 2	FL=90	3	**Mosunetuzumab IV** **(1/2/6/60/30mg)**	AHCT (21)/CAR-T (3)	8 (8-18)	72	54	–	Neutropenia: 27, Hypophosphatemia: 17, Hyperglycaemia: 8, Anaemia: 8, CRS:1	NCT02500407 CRS most common AE, confined to cycle 1. Median PFS: 17.9 m
Budde et al (27)	2022	Phase 1/2 (Abs)	FL=12	3.5	**Mosunetuzumab subcutaneous**	NR	5 (1-17)	82	64	–	Neutropenia: 12, Serious infection: 8	NCT02500407 Activity is similar to intravenous
Morschhauser et al (28)	2022	Phase 1 (Abs)	FL=27	1	**Mosunetuzumab IV + Lenalidomide, 12 cycles**	NR	NR	92	77	15	Grade III-IV 30%	NCT04246086 MC AE CRS No TAE discontinuation
Morschhauser et al (29)	2021	Phase 1/2 (Abs)	FL=53	3	**Glofitamab IV monotherapy**	NR	NR	81	70	–	NR	MC AE CRS, N=1 Gr3 Neutropenia=26%
Morschhauser et al (29)	2021	Phase 1/2 (Abs)	FL=19	2	**Glofitamab IV + Obinutuzumab**	NR	NR	100	73.7	–	NR	Neutropenia=58% Anaemia=37% Thrombocytopenia=32%
Ghosh et al. (30)	2021	Phase 1b (Abs)	FL=23, tFL=6, MZL=1, MCL=1	2	**Glofitamab IV + RCHOP**	NR	NR	90	77	–	Grade 3: 90 CRS:9.7, Neurologic:13	NCT03467373
Phillips et al (31)	2022	Phase 1/2 (Abs)	MCL=37	3 (BTKi +)	**Glofitamab IV + Obinutuzumab pre-treatment**	NR	NR	83.8	73	–	Obinutuzumab 1000mg: CRS 25%, 2000mg: 9.5%	MC AE CRS and neutropenia No TAE discontinuation
Hutchings et al (32)	2021	Phase 1/2	FL=12	4.5	**Epcoritamab sub-cut monotherapy**	AHCT (8)/0	NR	90	50	–	None	MC AE CRS, Skin reaction, No TAE discontinuations
Merryman et al (33)	2023	Phase 1/2 (Abs)	FL=109	1	**Epcoritamab sub-cut + Rituximab + Lenalidomide, 12 cycles**	NR/CAR-T (n=2)	NR	97 POD24 = 98	86 POD24 = 75	–	CRS=3	MC AE CRS, neutropenia and skin site reaction. POD24, CR in 2L: 90% Estimated 6m PFS: 93%
Patel et al (34)	2022	Phase 1	Mixed (n=25, FL=6)	4	**Plamotamab IV**	NR/CAR-T(FL=0)	NR	100	50	–	Anaemia=19, Neutropenia=16 thrombocytopenia=10	MC AE CRS, none grade 3. TAE discontinuation 13
Banerji et al (21)	2022	Phase 1 (Abs)	FL=40, MCL=12, MZL=6 (DLBCL=85)	3	**Odronextamab IV**	AHCT (8)/CAR-T (29)	NR	FL=91 MCL=50, MZL=67	FL=72, MCL=33, MZL=33	FL=15 MCL=17 MZL=33	anaemia 25, lymphopenia 19, hypophosphataemia 19, neutropenia 19, thrombocytopenia 14, CRS 7	MC AE CRS 48% treatment interruption or delay RP2D FL, 80mg FL, Median PFS= 17.1m
Novelli et al (35)	2023	Phase-2 ELM-2 study (Abs)	FL=131	3	**Odronextamab IV**	NR		82	75		CRS (n=1)	Median F/U: 20.5m, Median PFS=20.2m MC AE CRS, Neutropenia
Budde et al (36)	2021	Phase 1 (Abs)	FL=13, MCL=3, MZL=2 [DLBCL=11]	3	**IGM 2323 (Imvotamab), IV**	AHCT (2)/CAR-T (7)	Range= 1-42	Fl, MZL =33 MCL=NR	NR	NR	Grade 5 AE N=3 CRS, n=1	MC AE Hypophosphatemia, CRS
Hou et al. (37)	2022	Phase 1 (Abs)	FL=12, MZL=4, MCL=4 [DLBCL=7]	4	**TNB-486, IV**	AHCT (15)/CAR-T(19)	NR	FL=88 (7/8pts) MZL=75 (3/4)	FL=88, MZL=75		Lymphopenia=26, Neutropenia=15, Neurotoxicity=7	MC AE CRS, none grade 3, only in cycle 1.
Nair et al. (38)	2023	Phase 1 (Abs)	FL=17	3	**TNB-486, IV**	AHCT (6)/CAR-T (12)/CD20-TCE (12)	7 months	FL (n=11), 91	91		Lymphopenia 35, Neutropenia 12, ICANS (n=1)	6m PFS=91% High response rates in POD24, CAR-T treated, CD20 negative
Yang et al. (39)	2023	Phase 1/2 (Abs)	42, mixed B-NHL	3	**IMM0306, IV**	NR	NR	FL=36 (4/11)	FL (2/11)	NR	Lymphopenia=59.5, Neutropenia=11.9 Thrombocytopenia (1), Diarrhoea (1)	MC AE Lymphopenia, Leucopenia, Anaemia, neutropenia, thrombocytopenia
Hutchings et al. (40)	2023	Phase-1(Abs)	Indolent(i) B-NHL =23 Aggressive NHL= 57	3	**RO7227166 + Glofitamab, IV**	AHCT(NR)/CAR-T(4)	NR	iNHL = 95.7	iNHL = 74	NR	Study related grade 5, n=2, CRS n=1,	MC AE CRS, Neutropenia, Anaemia
Wang et al. (41)	2023	Phase 1 (Abs)	B-NHL and B-ALL	–	**GNC-038 (Emfizatamab)**	–	–	–	–	–		NCT04606433

### Mosunetuzumab

An IgG-like CD20xCD3 BsA, is the first-in-its class of BsA and underwent an extensive period of pre-clinical development. In a phase 1/2 monotherapy clinical trial in patients with RR indolent B-cell lymphomas, a majority of whom had received at least 3 lines of prior therapy including CAR-T, the complete response (CR) rates, duration of response (DoR) and median progression-free survival (PFS) were an impressive 48.5%, 16.8, and 11.8 months respectively (25). In the FL subset from the same trial, the outcomes were 54%, 22.8 months, and 17.9 months, respectively (26). The median age of this patient cohort (n=90) was 60years, with nearly 70% refractory to previous anti-CD20 therapy, 53% double refractory to anti-CD20 and alkylating agent based prior treatments, and 52% belonging to the POD24 (progression of disease within 24 months) subset. Administered in 21 day cycles, patients received a step-up dosing schedule in the first cycle with 1mg on day 1, 2mg on day 8 and 60mg on day 15. In cycle 2, patients received 60mg on day 1 and in subsequent cycles thereafter, received a fixed dose of 30mg on day 1. In patients who achieved a CR, treatment was completed after cycle 8, while in the partial responders treatment was continued up to 17 cycles. Retreatment was allowed in patients who progressed after achieving CR. A smaller study (n=12) using Mosunetzumab, as a subcutaneous formulation, in heavily pre-treated patients with RR Follicular lymphoma demonstrated similar outcomes as reported in the intravenous formulation (27). Mosunetuzumab has also been tested in a combinatorial regimen with Lenalidomide in patients with RR FL (n=27). The overall response rate (ORR) and CR were 92% and 77%, respectively (28).

### Glofitamab

Is a IgG-like CD20xCD3 BsA which differs from Mosunetuzumab in possessing two CD20 binding sites in a 2:1 format, and this potentially translates into a 40-fold increase in tumour cell lysis, based on an *in-vitro* study (42). In a phase 1/2 study in RR B-cell lymphomas, patient cohorts were administered Glofitamab in a step-up dosing regimen alone and in combination with one dose of Obinutuzumab as prephase (43). An analysis of patients with RR FL reported an ORR and CR of 81% and 70% respectively in the monotherapy cohort, while outcomes in the Obinutuzumab combination cohort were 100% and 73.7% respectively, suggesting similar CR rates (29). In the single-arm pivotal Phase II expansion in patients with RR DLBCL and 2 or more prior lines of therapy, 155 patients received glofitamab with initial step-up dosing, obinutuzumab pre-treatment and a fixed duration of therapy for a maximum of 12 cycles (each of 21 days) (44). The ORR was 52% with 39% of patients reaching CR. Responses were observe across all large cell/high grade B-cell lymphoma histologies and those patients that had relapsed after prior CAR-T therapy. Complete responses have proven to be highly durable (45). In patients with RR MCL who had received a median of three prior lines of therapy including a Bruton-tyrosine kinase inhibitor (BTKi), Glofitamab was administered in a step-up dosing after pre-treatment with one dose of Obinutuzumab. The ORR and CR were 83.8% and 73%, respectively (31). Glofitamab in combination with RCHOP was administered in a phase-1b study in patients with RR indolent B-cell lymphomas, a majority of whom were FL, and a median of 2 prior lines of therapy. The ORR and CR were reported in an abstract form as 90% and 77% respectively, while the toxicity was considered acceptable (30).

### Epcoritamab

Is a subcutaneously administered IgG-like CD20xCD3 BsA. In the phase-1/2 dose-escalation study (0·0128–60 mg), 12 patients with RR FL received Epcoritamab monotherapy, after a median of 4.5 prior lines of therapy, and demonstrated an ORR of 90% and CR of 50% with no grade 3 or 4 adverse events (32). The recommended phase 2 dose was 48mg. A combination regimen of subcutaneous epcoritamab, rituximab and lenalidomide was studied in a phase 1/2 study in patients with RR FL, in the EPCORE NHL-2 trial (NCT04663347). A pooled analysis from cohorts 2a and 2b reported an impressive 97% ORR and 86% CR, in this patient group who had received a median of at least one prior line of therapy. Even in the subset of POD24 patients, the ORR and CR rates were promising at 98% and 75%, improving to a CR rate 90% if the regimen was administered as second line therapy (33). This regimen will now proceed to a phase 3 study, NCT05409066. In the EPOCRE NHL-1 large B-cell lymphoma expansion study, 157 patients were treated who had received 2 or more prior lines of therapy. Step-up dosing was used to mitigate toxicity and therapy was given until disease progression (46). The OOR was 63% with 39% of patients achieving CR. Responses were similar across all sub-groups including the number of prior lines of therapy and previous CAR-T exposure. As observed with glofitamab in a similar patient population the CRs have proven to be durable.

### Plamotamab

Is also an IgG-like CD20xCD3 BsA which has been recently evaluated in a multicentre phase-1 study in a heavily pre-treated cohort of patients with RR B-cell non-Hodgkin lymphomas. All the patients with FL (n=6) responded, with a 50% CR (34). *Odronextamab*, an IgG-like CD20xCD3 BsA, has been studied in a large dose-finding phase-1 study [with FL=40, MCL=12, marginal zone lymphoma (MZL)=6], and reported impressive ORR of 91%, 50% and 67% in FL, MCL and MZL respectively. The CR rates were respectively, 72%, 33% and 33% (21). The dose for phase-2 follicular lymphomas was determined to be 80mg. In a large phase-2 study, Odronextamab maintained the response rates at a high of 82% ORR and 75% CR in patients with FL who had received a median of three prior lines of treatment (35). The treatment was well tolerated.

### Imvotamab

(IGM-2323) is an IgM-like CD20xCD3 BsA with a modified J chain (in the IgM structure) holding CD20xCD3 in a 10:1 molar ratio (47).A phase-1 study recently reported 33% ORR in the subset of heavily pre-treated patients (n=15) with FL and MZL (36).

### TNB-486

Is an IgG-like CD19xCD3 BsA and has been evaluated in a phase 1 study of patients with RR B-cell lymphomas with a median of 4 prior lines of failed treatments, which included CAR-T and transplant in a majority. The CR rate in patients with FL and MZL were 88% and 75% respectively (37). An update from the cohort of FL reported promising outcomes in this group of patients, who included POD24, CAR-T treated and CD20 negative tumours, with 91% CR rates (38). 

*IMMO306* is an CD20xSIRPα(CD47) BsA which links up with the CD47 receptor on macrophages and NK cells and activates them, by the blockade of CD47-SIRPα interaction, accompanied by FcɣR engagement. These interactions result in the phagocytosis of the tumour cell (48). In an ongoing phase 1 study of patients with RR B-cell lymphomas, IMMO306 demonstrated an ORR of 36% in patients with RR FL (39). RO7227166 (*Englumafusp alpha*) is an IgG like CD19x4-1BB fusion protein which targets the co-stimulatory domain, 4-1BB (CD137), on T-cells as well as CD19 on B-cells. It has been studied in a phase-1 study of patients with RR B-cell Lymphomas in combination with Glofitamab. The cohort of patients with indolent lymphomas (n=23) demonstrated an ORR of 95.7% and CR of 74%, without significant grade 3 or 4 toxicities (40). *GNC-038*, an IgG-scFv-conjugated tetra-specific antibody targeting αCD19xCD3x4–1BBxPD-L1, is being evaluated in an ongoing phase-1 study in RR B-cell lymphomas and B-cell lymphoblastic leukaemia (41).

## Adverse events and management strategies

Outcomes from early phase studies suggest a predictable and manageable toxicity profile with the use of BsA. These are attributable primarily to the consequences of immune cell (T-cell) overactivation, ‘on-target off-tumour’ effects, and tumour cell lysis. The key adverse events are cytokine release syndrome (CRS), neurotoxicity, infections and tumour lysis syndrome(s). The mechanisms underlying and a more extensive review can be found elsewhere (49, 50). In the monotherapy studies, these events have been mostly grade 1 and grade 2 in nature, with a majority (up to 90%) of patients developing CRS and less commonly, neurotoxicity. The incidence of greater than grade 3 toxicity have <10% in these early phase studies. **Table 2** lists the incidence and comments on the adverse events in individual studies. A tumour flare reaction has also been observed in many of these studies. The consensus criteria developed for CAR-T therapies for CRS and neurotoxicity grading and management are applied to BsA too, though they may not exactly overlap in their mechanisms (51). CRS is observed in the initial cycles, usually in cycle 1 and presents with fever and chills, skin rash, hypotension and confusion.

Neurotoxicity syndromes commonly present as headache, tremors, agitation, lethargy, difficulty in concentration, confusion, delirium, etc. and very rarely manifests in more severe forms like aphasia, ataxia, akinesia, myoclonus, encephalopathy or seizures. In the pivotal study with Mosunetuzumab, the incidence of the neurotoxicity syndromes were <3% and predominantly grade 1-2 (26). These Immune-effector cell-associated neurotoxicity syndromes (ICANS) typically develop in the first week, and seldom occur beyond cycle 1. They need close and serial clinical monitoring, neuroimaging and EEG. The management is largely supportive and preventative, and pharmacological treatment involves high dose corticosteroids. It is increasingly rare to see high grade ICANS with the usage of step-up dosing schedules of BsA (52). Tumour lysis syndrome is rare and this is attributed to the use of adequate hydration and hypouricemic agents prophylactically, and is commoner in patients who have a background of renal dysfunction. Death directly attributable to the BsA in indolent B-cell lymphomas have not been significant, nearly none, with only 2 studies reporting them till date (21, 25). Mitigation strategies used in most of these studies (**Table 2**) have included the use of step-up dosing, mandatory hospitalisation and pre-phase cytoreductive strategies (e.g. Obinutuzumab).

Patients receiving BsA must be monitored closely for infections as they are at increased risk of viral, bacterial and occasionally fungal infections due to an immunosuppressive environment. Beyond the cancer and the effects of prior treatments, BsA related cytopenias, ‘off-tumour on-target’ effect of B-cell hypoplasia, and T-cell exhaustion lead to significant immunosuppression (53). Close monitoring to include evaluation of rare opportunistic infections, growth factor support, anti-microbial prophylaxis (e.g. for herpes virus and pneumocytis jiroveci), and immunoglobulin replacement are mitigation strategies used to prevent them. With regards to SARS-COV2, the risk of infection, hospitalisation and poor outcomes are high (54). The use of RNA vaccinations and treatment with monoclonal antibodies during infections should be considered in these patients.

Apart from the aforementioned adverse events, the other side-effects of note are neutropenia, anaemia, lymphopenia, hypophosphatemia, fatigue and diarrhoea. In the studies, listed in **Table 2**, the incidence of these toxicities vary between 15-45% and are usually mild (grade 1-2). With experience and long-term safety data, we will better understand the use of BsA and potentially manage the adverse events more efficiently.

## Considerations for the use of BsA and challenges thereof

We are at an interesting time point in the evolution of therapies for lymphomas after the advent of molecular diagnostics, immunotherapies and targeted therapies. Small molecule inhibitors, and immunotherapies such as monoclonal antibodies, BsA, and CAR-T therapies have steadily improved outcomes and are pushing the treatment paradigm in the direction of chemotherapy-free regimens. In the context of indolent lymphoproliferative disorders, chronic lymphocytic leukaemia management is a case in point (55). In the following section, we will attempt to summarise some key takeaways and challenges in indolent B-cell Lymphomas.

1. The use of BsA will further augment the treatment armamentarium of relapsed and refractory indolent b-cell lymphomas by improving outcomes in a manner hitherto unimaginable, with predictable and manageable acute adverse events. The premise of a manufactured-to-scale, ready-to-use, off-the-shelf drug, with a potential for a short learning curve and easy adoption in community oncology settings makes it a compelling candidate for change.
2. The duration of therapy and mode of administration of treatment are important determinants for wide acceptance of therapy. Patients and physicians are disinclined towards adopting treatments which require frequent hospital visits and closer monitoring. While Glofitamab and Mosunetuzumab have been tested in fixed duration schedules, epcoritamab and odronextamab are given until progression or toxicity in the ongoing trials. Further, the current group of BsA are all intravenously administered, with the exception of epcoritamab (and mosunetuzumab) which has subcutaneous formulation. A clear preference for subcutaneously administered treatment exists given the benefit in logistics of care delivery.
3. The paucity of long term data and real-world evidence with BsA are challenges only time will overcome. Long term survival and toxicity data, and efficient management of adverse events are key to the eventual establishment of BsA as standard of care. Further studies on the predictors of systemic immune response and toxicity are required to help better understand this therapy.
4. The role of CAR-T therapy in indolent B-cell lymphomas is under evaluation in multiple clinical trials and their results will potentially establish its role in this setting (56). Tisagenlecleucel was granted approval by US-FDA in 2022 in RR FL, after the failure of 2 or more lines of treatment (57). The benefits and disadvantages of CAR-T therapy over BsA in the management of lymphomas is an ongoing debate and has been argued well elsewhere (58).

## Future directions

1. There is a panoply of targeted therapies and immunotherapeutic agents in indolent lymphomas today, with few or no studies directly comparing these agents in randomised clinical trials. Cross-trial comparisons would not be an ideal tool to determine which BsA (or immunotherapeutic agent) outshines the rest. Going forward, this limitation in data is expected to be addressed. Ongoing trials reported in **Table 2** represent the first wave of clinical trials. Combinatorial regimens harnessing multi-targeted approaches such as Epcoritamab, Rituximab and Lenalidomide (33) which appears promising even in the POD24 group of FL, will see progress. Similar regimens are being tested in ongoing studies, some noteworthy trials are Mosunetuzumab with Polatuzumab vedotin and Obinutuzumab (NCT05169658) in untreated indolent B-cell lymphomas, Tazemetostat (EZH2 inhibitor) with Mosunetuzumab (NCT05994235) in newly diagnosed follicular lymphoma, Glofitamab with Obinutuzumab (NCT05783596) in untreated follicular lymphoma, and Mosunetuzumab with Lenalidomide in relapsed follicular lymphoma (NCT04712097)/relapsed Marginal zone lymphoma (NCT06006117).
2. Resistance to BsA therapy is known and there is increasingly an understanding that this may be driven primarily by the loss of target antigen by tumour cells, apart from T-cell exhaustion/dysfunction, and multiple cellular and humoral factors present within the tumour microenvironment (49). Determination of biomarkers for resistance, and the interplay between different tumour-immune cascades is driving the next stage of development in this therapy; multi-specific antibody therapies, fusion proteins, probody therapies, etc. are worthy of note (59, 60). An example of such an approach is an ongoing clinical study is evaluating the signatures of response and resistance to treatment with Mosunetuzumab (NCT05529524).
3. Finally, as BsA and other novel therapies advance quickly from the RR setting to first line therapy, the tough questions to answer would be the selection, optimal use (alone or in combination), and sequencing of therapies. While fixed duration regimens such as NCT05783596 using Glofitamab and Obinutuzumab are hoping to deepen responses in patients with indolent lymphomas, trials such as NCT04889716 (CAR-T therapy followed by BsA) are testing the role of sequential treatment strategies.

## Author contributions

VR: Writing – original draft, Writing – review & editing, Conceptualization, Data curation. AD: Conceptualization, Writing – review & editing, Resources.
